# Diagnostic value of the urine lipoarabinomannan assay in HIV-positive, ambulatory patients with CD4 below 200 cells/μl in 2 low-resource settings: A prospective observational study

**DOI:** 10.1371/journal.pmed.1002792

**Published:** 2019-04-30

**Authors:** Helena Huerga, Sekai Chenai Mathabire Rucker, Loide Cossa, Mathieu Bastard, Isabel Amoros, Ivan Manhiça, Kuzani Mbendera, Alex Telnov, Elisabeth Szumilin, Elisabeth Sanchez-Padilla, Lucas Molfino

**Affiliations:** 1 Epicentre, Paris, France; 2 Médecins Sans Frontières, Lilongwe, Malawi; 3 Médecins Sans Frontières, Maputo, Mozambique; 4 Ministry of Health, Maputo, Mozambique; 5 Ministry of Health, Lilongwe, Malawi; 6 Médecins Sans Frontières, Geneva, Switzerland; 7 Médecins Sans Frontières, Paris, France; FIND, SWITZERLAND

## Abstract

**Background:**

Current guidelines recommend the use of the lateral flow urine lipoarabinomannan assay (LAM) in HIV-positive, ambulatory patients with signs and symptoms of tuberculosis (TB) only if they are seriously ill or have CD4 count ≤ 100 cells/μl. We assessed the diagnostic yield of including LAM in TB diagnostic algorithms in HIV-positive, ambulatory patients with CD4 < 200 cells/μl, as well as the risk of mortality in LAM-positive patients who were not diagnosed using other diagnostic tools and not treated for TB.

**Methods and findings:**

We conducted a prospective observational study including HIV-positive adult patients with signs and symptoms of TB and CD4 < 200 cells/μl attending 6 health facilities in Malawi and Mozambique. Patients were included consecutively from 18 September 2015 to 27 October 2016 in Malawi and from 3 December 2014 to 22 August 2016 in Mozambique. All patients had a clinical exam and LAM, chest X-ray, sputum microscopy, and Xpert MTB/RIF assay (Xpert) requested. Culture in sputum was done for a subset of patients. The diagnostic yield was defined as the proportion of patients with a positive assay result among those with laboratory-confirmed TB. For the 456 patients included in the study, the median age was 36 years (IQR 31–43) and the median CD4 count was 50 cells/μl (IQR 21–108). Forty-five percent (205/456) of the patients had laboratory-confirmed TB. The diagnostic yields of LAM, microscopy, and Xpert were 82.4% (169/205), 33.7% (69/205), and 40.0% (84/205), respectively. In total, 50.2% (103/205) of the patients with laboratory-confirmed TB were diagnosed only through LAM. Overall, the use of LAM in diagnostic algorithms increased the yield of algorithms with microscopy and with Xpert by 38.0% (78/205) and 34.6% (71/205), respectively, and, specifically among patients with CD4 100–199 cells/μl, by 27.5% (14/51) and 29.4% (15/51), respectively. LAM-positive patients not diagnosed through other tools and not treated for TB had a significantly higher risk of mortality than LAM-positive patients who received treatment (adjusted risk ratio 2.57, 95% CI 1.27–5.19, *p =* 0.009). Although the TB diagnostic conditions in the study sites were similar to those in other resource-limited settings, the added value of LAM may depend on the availability of microscopy or Xpert results.

**Conclusions:**

LAM has diagnostic value for identifying TB in HIV-positive patients with signs and symptoms of TB and advanced immunodeficiency, including those with a CD4 count of 100–199 cells/μl. In this study, the use of LAM enabled the diagnosis of TB in half of the patients with confirmed TB disease; without LAM, these patients would have been missed. The rapid identification and treatment of TB enabled by LAM may decrease overall mortality risk for these patients.

## Abstract 2

Helena Huerga and colleagues assess a urine-based lipoarabinomannan assay for diagnosing tuberculosis in immunocompromised patients with HIV.

## Author summary

**Why was this study done?:**

- Tuberculosis (TB) is the leading cause of death in HIV-positive patients, but it remains difficult to diagnose. The availability of diagnostic tests is limited, and most require sputum samples, which are difficult to obtain from very sick patients.
- The lipoarabinomannan assay (LAM) is a urine-based point-of-care test that has shown utility in immunosuppressed HIV-positive patients. However, this test is not widely used.
- Current international guidelines recommend the use of the LAM test only in extremely immunocompromised HIV-positive, ambulatory patients. However, we hypothesized that the test could also be useful to diagnose TB in a broader group including less severely immunocompromised ambulatory patients.

**What did the researchers do and find?:**

- We conducted a prospective observational study in 6 health facilities in Malawi and Mozambique, enrolling HIV-positive, ambulatory patients with symptoms of TB. The clinicians conducted a clinical exam, requested diagnostic tests for TB (LAM in urine, microscopy, Xpert MTB/RIF assay [Xpert], culture in sputum, and chest radiography) at the first consultation, and followed the patients at subsequent visits over a 6-month period.
- Of the 456 enrolled patients, 205 (45%) had TB; of these, LAM was positive in 82.4% (169/205), microscopy in 33.7% (69/205), and Xpert in 40.0% (84/205).
- Using LAM in addition to the other available diagnostic tools increased the proportion of patients diagnosed with TB by 38.0% compared to a diagnostic algorithm including clinical exam, chest X-ray, and microscopy, and by 34.6% compared to an algorithm including clinical exam, chest X-ray, and Xpert. A similar increase was observed for less severely immunocompromised patients.

**What do these findings mean?:**

- The use of the urine-based LAM test can increase the proportion of immunocompromised HIV-positive patients diagnosed with TB, including patients who are less severely immunocompromised.
- LAM is useful in a broader population than what it is currently recommended for, and can be expanded for use in patients who are less severely immunocompromised.

## Introduction

Tuberculosis (TB) is the leading cause of death in HIV-positive individuals [1]. Progressive immunosuppression is associated with disseminated disease and high mortality risk, as well as more severe forms of TB. Despite their poor sensitivities, clinical signs, sputum microscopy, and X-ray are commonly used tools for TB diagnosis in resource-limited settings. Although the Xpert MTB/RIF assay (Xpert) performs well as a diagnostic tool, in many settings it is either not at all available or not available as a point-of-care tool. Moreover, very sick patients may struggle to provide sputum samples for microbiological testing, leading to missed TB diagnoses and delays in TB treatment initiation.

The lipoarabinomannan assay (LAM) (Alere Determine TB-LAM Ag lateral flow strip) is a point-of-care test that detects the presence of the *Mycobacterium tuberculosis* (MTB) cell wall antigen lipoarabinomannan in urine and provides results within 25 minutes [2]. LAM diagnoses TB in HIV-positive patients with advanced immunodeficiency in whom the antigen enters the urine primarily by haematogenous TB dissemination to the kidneys [3,4]. The sensitivity of the assay depends on the level of immunosuppression (i.e., CD4 count). In a meta-analysis of 5 studies including patients with CD4 ≤ 200 cells/μl, the pooled sensitivity and specificity were 50% and 90%, respectively [5]. This low accuracy may in part reflect a poor diagnostic reference standard. Indeed, the specificity of LAM increases considerably when the reference standard includes several body samples in addition to sputum [6]. Testing on urine samples increases diagnostic feasibility for HIV-infected patients who are unable to produce sputum [7,8]. In addition, previous studies have shown that including LAM in TB diagnostic algorithms is cost-effective [9,10]. Importantly, LAM also serves to identify patients at a higher risk of mortality [11–14]. A randomised multicentre trial has shown that, compared to the standard of care, the use of LAM in hospitalised patients with at least 1 symptom of TB reduced mortality by 17% (95% CI 4%–28%) [15]. In another randomised multicentre trial, a decreased mortality was observed in patients who received a LAM test compared to those who did not among patients with clinical suspicion of TB [16].

In 2015, based on the evidence available at the time, WHO released a policy guideline on the use of LAM stating that the test may be used to assist in the diagnosis of TB in HIV-positive adults with signs and symptoms of TB who are seriously ill or have a CD4 count less than or equal to 100 cells/μl [17]. Despite this recommendation, most of the national TB programmes in African countries are not currently using the test to diagnose TB [18]. More evidence on the use of LAM in programmatic conditions would help inform programme managers on how to use this test in their practice, and how best to incorporate it into national algorithms. Research also suggests that LAM may be valuable for ambulatory patients with signs and symptoms of TB and a CD4 count below 200 cells/μl, and not only for those with CD4 count below 100 cells/μl [19,20].

This study assessed the diagnostic yield of including LAM in TB diagnostic algorithms in HIV-positive patients with signs and symptoms of TB and CD4 < 200 cells/μl. In addition, we assessed the risk of mortality in patients with a LAM-positive result who were not diagnosed using other diagnostic tools and who were not treated for TB.

## Methods

### Study design and study population

We conducted a prospective observational study in 6 health facilities in Malawi and Mozambique (S1 Appendix). The study protocol and analysis plan were approved by the Médecins Sans Frontières Ethics Review Board and by the national ethical review committees from Malawi and Mozambique (S2 Appendix). Patients were consecutively included from 18 September 2015 to 27 October 2016 in Malawi and from 3 December 2014 to 22 August 2016 in Mozambique. The study population included HIV-positive patients ≥15 years old with a CD4 count of less than 200 cells/μl presenting with at least 1 of the following symptoms of TB: fever, cough, night sweats, or self-reported weight loss, who sought care at one of the study sites and provided written voluntary informed consent. Patients were excluded if they had taken fluoroquinolones or anti-TB drugs in the previous month. Patients presented for general care consultations or for new or follow-up HIV visits to one of the following health facilities: the out-patient facility of Chiradzulu District Hospital or Namitambo, Mirepa, or Mauwa health centre in Chiradzulu District, Malawi, or Alto Maé or Primeiro de Maio health centre in Maputo, Mozambique. These facilities were run by the Ministry of Health of each country and were supported by Médecins Sans Frontières for HIV and TB care.

### Study procedures

At arrival, patients were seen by a nurse or a clinical officer who assessed the patient with regard to the study inclusion and exclusion criteria and initiated the informed consent procedures for those eligible. Written informed consent was obtained from all participants. For patients under 18 years old, consent was obtained from at least 1 parent/legal guardian and assent was obtained from the patient. Once the informed consent was provided, patients were seen by a medical doctor or a clinical officer who conducted a clinical examination and requested urine and spontaneously expectorated sputum (1 on spot and 1 early morning) samples. The non-provision of a urine or sputum sample was not an exclusion criterion. Urine was tested with the Alere Determine TB-LAM Ag assay (Alere, Waltham, MA, US) on the same day by applying 60 μl of fresh urine to the sample pad on the assay strip. Tests were interpreted following the manufacturer’s instructions using grade 1 (lowest of 4 grades) as the cutoff point for a positive result. Smear microscopy was performed on unprocessed sputum at the health centres using auramine staining and light-emitting diode fluorescence microscopy. Patients with at least 1 smear with more than 1 acid-fast bacillus per 100 high-power fields were considered smear positive. Sputum samples were transported from the health centres to the laboratory and were also tested using Xpert (Cepheid, Sunnyvale, CA, US). In Malawi, Xpert was performed in the laboratory of Chiradzulu District Hospital and, in Mozambique, at the National Reference Laboratory of Tuberculosis in Maputo. The test was repeated using a new cartridge in case of “invalid”, “error”, or “no result” on the first sample. Xpert results were defined as positive when MTB was “detected”, negative when MTB was “not detected”, and “not available” when the test was not performed or the results were reported as “invalid”, “error”, or “no result” on at least 1 of the 2 tests. In addition, in Maputo, mycobacterial culture on sputum was done in liquid (MGIT) and solid (Lowenstein Jensen) medium at the national reference laboratory. A posterior–anterior chest X-ray was requested for all patients at the initial consultation. However, the X-ray was often not done on the same day, particularly in Malawi, due to the distance from the health centres to the district hospital, where X-rays were performed, and due to the fact that X-ray was not available on all days. In the absence of clinical or radiological findings suggestive of TB, patients were prescribed a broad-spectrum antibiotic for 1 week and were reassessed 5 days after the first consultation. The treating clinicians decided whether or not to start TB treatment at any time during this process based on their clinical assessment, the biological test results, and the chest X-ray findings, according to the national guidelines for TB diagnosis and treatment. In Malawi, during the first 9 months of recruitment, LAM test results were not used for patient management as per the request of the Malawi National Tuberculosis Control Programme. Therefore, study clinicians were blinded to the LAM results. During this time, LAM was done in the hospital laboratory. Once permission was obtained to use the results of the LAM test for patient management, the clinicians started performing the test themselves during patient consultations. In Mozambique, the results of the LAM tests were used by the clinicians from the beginning of the study. All necessary TB and HIV care, including clinical follow-up, CD4 monitoring, ART initiation, counselling, and other laboratory investigations, was provided free of cost. All patients, whether started on TB treatment or not, were followed for 6 months. When patients did not attend scheduled visits, they were traced either by a phone call or home visit.

### Statistical analyses

We defined laboratory-confirmed TB (definite TB) as at least 1 positive laboratory test, whether LAM, smear microscopy, Xpert, or culture. To assess the diagnostic yield of the different assays, we calculated the proportion of patients with a positive assay result among those with laboratory-confirmed TB. In addition, we assessed the proportion of patients with a conclusive laboratory test result (positive or negative) among all patients. To assess the diagnostic yield of algorithms, we calculated the proportion of patients diagnosed through the standard-of-care algorithms used in the sites at the time of the study (clinical exam, microscopy or Xpert, and chest X-ray) with and without LAM among patients with laboratory-confirmed TB. The diagnostic yield was assessed from the perspective of the patient’s management, with the objective of replicating real-life decision-making.

Continuous variables were summarised as median and interquartile range (IQR). Categorical variables were summarised as counts and percentages. We used the Wilcoxon rank-sum test to compare continuous variables and the chi-squared test to compare categorical variables. All analyses were done independently in patients with CD4 < 100 cells/μl and in patients with CD4 100–199 cells/μl. Patients were considered seriously ill if declared as such by the clinician at the first consultation based on the patient having at least 1 of the following 4 danger signs: temperature higher than 39°C, respiratory rate higher than 30 respirations/minute, cardiac rate higher than 120 beats/minute, or inability to walk without help. In addition, in an a posteriori analysis, the proportion of patients defined as seriously ill was reassessed by adding the patients who fulfilled the definition based on recorded results of the clinical exam at the first consultation. Haemoglobin concentration (Hb) was used to define anaemia severity as severe (Hb < 80 g/l), moderate (Hb 80–119 g/l), or no anaemia (Hb ≥120 g/l). The time to test result was computed as the number of days between the day of the initial consultation and when the test result became available to the clinician.

We used a multivariable binomial regression model to assess the effect of not treating LAM-positive patients (as a surrogate of missing TB diagnosis by not using LAM) on mortality in the first 6 months from enrolment among patients with laboratory-confirmed TB. LAM-positive patients were not treated only during the first 9 months of recruitment in the Malawi site. We created a 4-category variable combining the LAM test result and the TB treatment decision as follows: LAM-positive and treated, LAM-positive and not treated, LAM-negative and treated, LAM-negative and not treated. We included the following variables in the analyses: age, sex, body mass index (BMI), antiretroviral therapy (ART), seriously ill as per clinical exam, CD4 count, and severe anaemia. We initiated the model with all the listed variables, and we selected the most parsimonious model using the Akaike Information Criterion (AIC) as the final multivariable model. The only variable of interest that had missing data was anaemia. AIC and Bayesian Information Criterion (BIC) were calculated for a complete case analysis excluding the missing values and including the missing values as a separate category (S3 Appendix). In addition, to assess the impact of missing values on the point estimates, we fitted a separate multivariable regression where missing values were imputed with multiple sequential imputation using chained equations (S4 Appendix). Risk ratios and 95% confidence intervals were assessed.

## Results

### Patient characteristics

**Fig 1:**
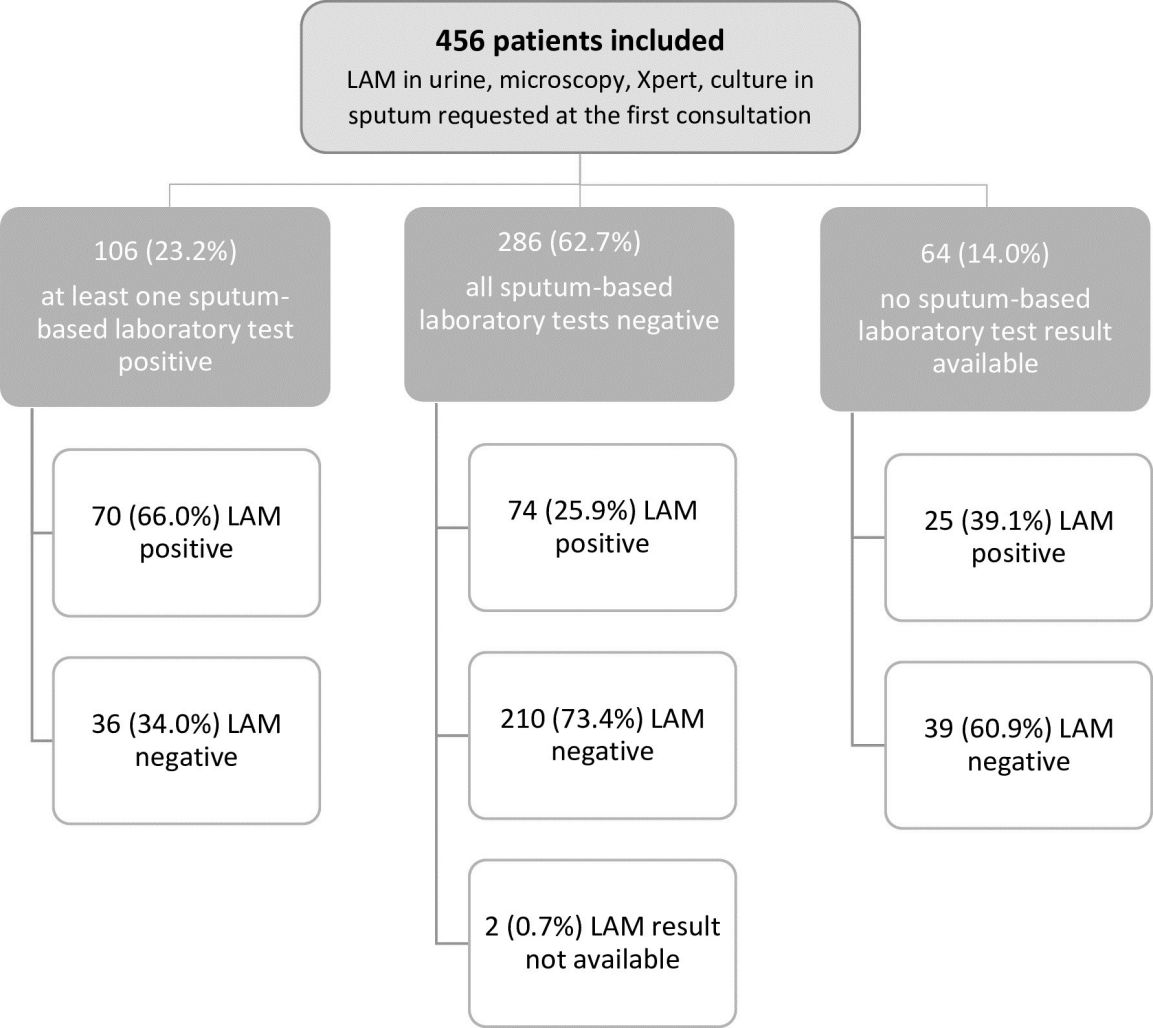
Patient flow and TB diagnostic test results. LAM, lipoarabinomannan assay; TB, tuberculosis; Xpert, Xpert MTB/RIF assay.

In total, 456 HIV-positive patients (285 in Mozambique and 171 in Malawi) with signs and symptoms of TB met the study inclusion criteria and were included in the study (Fig 1). Table 1 presents the demographic and clinical characteristics and the laboratory results of the patients: 202 (44.3%) were female, median age was 36 years (IQR 31–43), median CD4 count was 50 cells/μl (IQR 21–108), 242 (53.1%) were on ART at the initial consultation, and 101 (22.2%) were seriously ill. Among the 456 patients, 110 (24.1%) had a CD4 count of 100–199 cells/μl and were not seriously ill. Of the patients with a given diagnostic test result available, 18.0% (69/383), 23.9% (84/351), 24.9% (52/209), and 37.2% (169/454) had a positive result for microscopy, Xpert, culture, and LAM, respectively. In total, 45.0% (205/456) of the patients had laboratory-confirmed TB. The reasons for not having available diagnostic test results were related to programme issues (such as difficulties in the transport of samples from the peripheral centres to the laboratory, or the irregular presence of staff responsible for collecting samples and performing microscopy at the health centres in Malawi), difficulties in producing a sputum or a urine sample, and contaminated culture results. Compared to Malawi, in Mozambique a lower proportion of the patients were on ART (38.6% versus 78.2%, *p <* 0.001), median CD4 was lower (40 cells/μl versus 79 cells/μl, *p <* 0.001), a higher proportion of the patients were seriously ill (28.7% versus 11.1%, *p <* 0.001), and a higher proportion had severe anaemia (20.2% versus 17.7%, *p =* 0.014). However, median BMI was higher in Mozambique (19.3 kg/m^2^ versus 18.5 kg/m^2^, *p =* 0.003).

**Table 1:** Demographic and clinical characteristics of patients and laboratory results: Overall and by CD4 count group.

Characteristic	All patients (CD4 < 200 cells/μl), *N =* 456	Patients with CD4 < 100 cells/μl, *N =* 32	Patients with CD4 100–199 cells/μl, *N =* 129
**Sex**			
Male	254 (55.7)	182 (55.7)	72 (55.8)
Female	202 (44.3)	145 (44.3)	57 (44.2)
**Age (years)**	36 (31–43)	35 (31–42)	39 (33–49)
**BMI (kg/m**^**2**^**)**	18.8 (17.2–21.2)	18.7 (17.1–21.0)	19.8 (17.9–21.7)
**CD4 count (cells/μl)**	50 (21–108)	31 (14–57)	146 (118–172)
**On ART at first consultation**	241 (52.9)	158 (48.3)	83 (64.3)
**Reported symptoms**			
Cough	436 (95.6)	312 (95.4)	124 (96.1)
Fever	348 (76.3)	249 (76.2)	99 (76.7)
Chest pain	313 (68.6)	207 (63.3)	106 (82.2)
Haemoptysis	19 (4.2)	10 (3.1)	9 (7.0)
Difficulty breathing	231 (50.7)	161 (49.2)	70 (54.3)
Night sweats	337 (73.9)	237 (72.5)	100 (77.5)
Weight loss	344 (75.4)	243 (74.3)	101 (78.3)
**Clinical exam findings**			
Temperature ≥ 38.0°C	73 (16.1)	61 (18.8)	12 (9.3)
Respiratory rate > 20/min	170 (37.3)	142 (43.4)	28 (21.7)
Cardiac frequency > 100/min	119 (26.1)	99 (30.3)	20 (15.5)
**Seriously ill**			
Declared by clinician	52 (11.4)	40 (12.2)	12 (9.3)
Recalculated using information from the clinical exam	101 (22.2)	82 (25.1)	19 (14.7)
**Chest X-ray result**			
Highly suggestive of TB	127 (46.7)	90 (41.7)	37 (66.1)
Might suggest TB	101 (37.1)	87 (40.3)	14 (25.0)
Not suggestive of TB	44 (16.2)	39 (18.1)	5 (8.9)
*Missing*	*184*	*111*	*73*
**Anemia**			
Severe (Hb < 80 g/l)	71 (19.0)	55 (21.2)	16 (14.2)
Moderate (Hb 80–119 g/l)	222 (59.5)	164 (63.1)	58 (51.3)
No anemia (Hb ≥ 120 g/l)	80 (21.5)	41 (15.8)	39 (34.5)
*Missing*	*83*	*67*	*16*
**Unable to produce sputum**	64 (14.2)	48 (14.9)	16 (12.4)
**Unable to produce urine**	2 (0.4)	1 (0.3)	1 (0.8)
**Smear microscopy (sputum)**			
Positive	69 (18.0)	47 (17.3)	22 (19.8)
Negative	314 (82.0)	225 (82.7)	89 (80.2)
*Not done*	*73*	*55*	*18*
**Xpert MTB/RIF assay (sputum)**			
MTB detected	84 (23.9)	62 (24.0)	22 (23.7)
MTB not detected	267 (76.1)	196 (76.0)	71 (76.3)
*Not done/not interpretable*	*105*	*69*	*36*
**Culture (sputum)**			
Positive	52 (24.9)	42 (24.1)	10 (28.6)
Negative	131 (62.7)	110 (63.2)	21 (60.0)
Non-tuberculosis mycobacteria	26 (12.4)	22 (12.6)	4 (11.4)
*Not done/contaminated*	*247*	*153*	*94*
**LAM (urine)**			
Positive	169 (37.2)	133 (40.8)	36 (28.1)
Negative—no line	234 (51.5)	160 (49.1)	74 (57.8)
Negative—line lighter than 1	51 (11.2)	33 (10.1)	18 (14.1)
*Missing*	*2*	*1*	*1*
**Grade among patients with positive LAM**			
Grade 1	66 (39.3)	51 (38.6)	15 (41.7)
Grade 2	43 (25.6)	29 (22.0)	14 (38.9)
Grade 3	23 (13.7)	19 (14.4)	4 (11.1)
Grade 4	36 (21.4)	33 (25.0)	3 (8.3)
*Missing*	*1*	*1*	*0*
**Laboratory-confirmed TB**	205 (45.0)	154 (47.1)	51 (39.5)
**Treated for TB**	291 (63.8)	224 (68.5)	67 (51.9)
**Laboratory-confirmed TB and treated for TB**	185 (90.2)	140 (90.9)	45 (88.2)
**LAM result and TB treatment**			
Negative and treated	141 (31.1)	104 (31.9)	37 (28.9)
Positive and treated	149 (32.8)	119 (36.5)	30 (23.4)
Negative and not treated	144 (31.7)	89 (27.3)	55 (43.0)
Positive and not treated	20 (4.4)	14 (4.3)	6 (4.7)
*Missing*	*2*	*1*	*1*

Data given as *n* (percent) or median (IQR).

ART, antiretroviral therapy; BMI, body mass index; Hb, haemoglobin concentration; LAM, lipoarabinomannan assay; MTB, *Mycobacterium tuberculosis*; TB, tuberculosis.

### Diagnostic yield of LAM, microscopy, and Xpert

Of the 205 patients with laboratory-confirmed TB, 205 (100%) had a LAM result, 175 (81.4%) a microscopy result, and 166 (81.0%) an Xpert result. The median time to result was 0 (IQR 0–0) days for LAM, 5 (IQR 3–8) days for microscopy, and 5 (IQR 2–9) days for Xpert.

**Fig 2:**
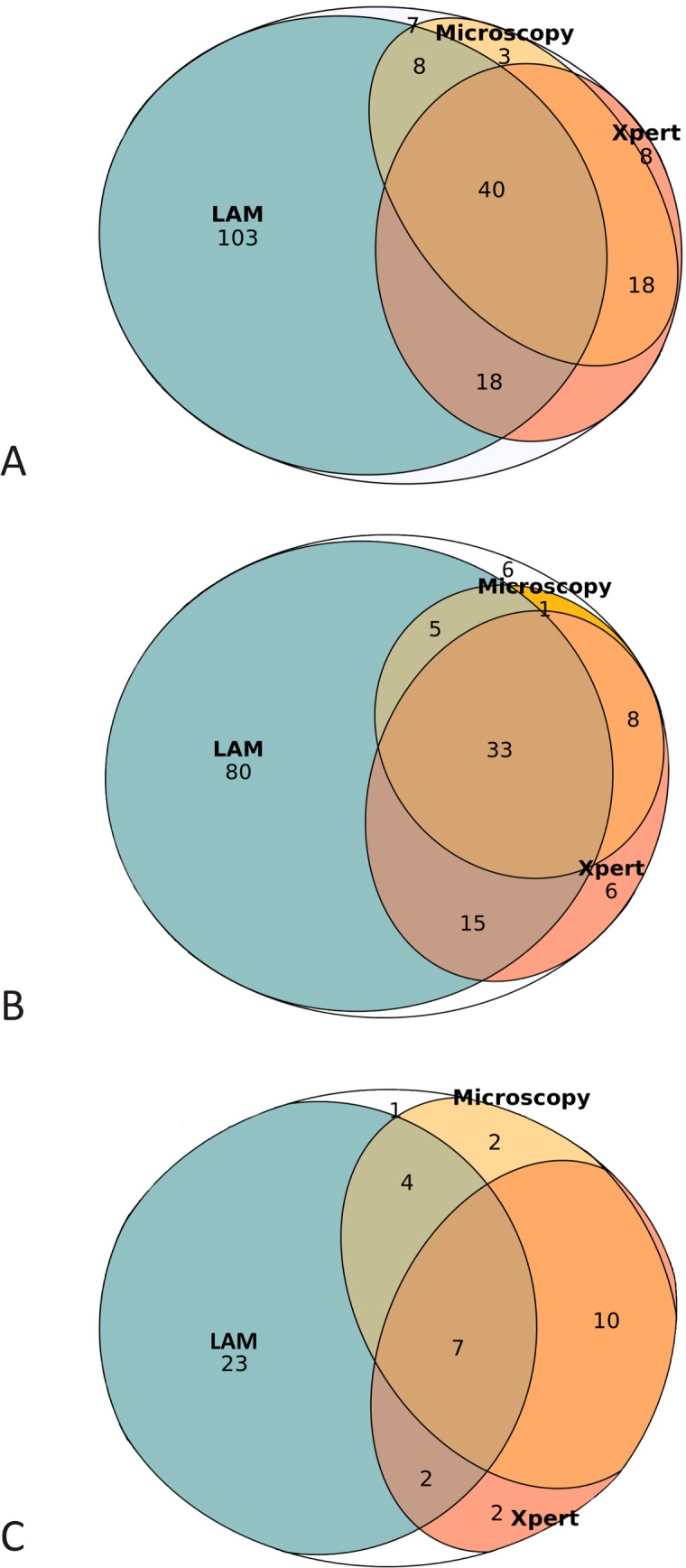
Laboratory-confirmed TB patients who would be diagnosed using LAM, microscopy, and Xpert. Euler diagrams showing the number of patients who would be diagnosed through LAM, microscopy, and Xpert among all laboratory-confirmed TB patients (A) and among laboratory-confirmed TB patients with CD4 < 100 cells/μl (B) and with CD4 100–199 cells/μl (C). Blank spaces show patients diagnosed solely with culture on sputum. The diagnostic yields of LAM, microscopy, and Xpert are shown in Table 2. LAM, lipoarabinomannan assay; TB, tuberculosis; Xpert, Xpert MTB/RIF assay.

Fig 2 shows the diagnostic yield of the 3 assays. The diagnostic yields of LAM, microscopy, and Xpert were 82.4% (169/205), 33.7% (69/205), and 41.0% (84/205), respectively. In patients with CD4 < 100 cells/μl, the diagnostic yields of LAM, microscopy, and Xpert were 86.4% (133/154), 19.5% (30/154), and 40.3% (62/154), respectively. In patients with CD4 100–199 cells/μl, the diagnostic yields were 70.6% (36/51), 43.1% (22/51), and 43.1% (22/51), respectively.

**Table 2:** Diagnostic yields of LAM, microscopy, and Xpert.

Laboratory test	All patients	Patients with CD4 < 100 cells/μl	Patients with CD4 100–199 cells/μl
LAM	82.4 (169/205)	86.4 (133/154)	70.6 (36/51)
Microscopy	33.7 (69/205)	19.5 (30/154)	43.1 (22/51)
Xpert	41.0 (84/205)	40.3 (62/154)	43.1 (22/51)

Data given as percent (*n/N*).

LAM, lipoarabinomannan assay; Xpert, Xpert MTB/RIF assay.

In total, 50.2% (103/205) of the patients with laboratory-confirmed TB were diagnosed only through LAM. Among patients with CD4 < 100 cells/μl and with CD4 100–199 cells/μl, 51.9% (80/154) and 45.1% (23/51), respectively, were diagnosed only through LAM. Microscopy and LAM in combination diagnosed 92.7% (190/205) of the patients with laboratory-confirmed TB, 59.0% more than microscopy alone. Xpert and LAM in combination diagnosed 95.1% (195/205) of the patients with laboratory-confirmed TB, 54.2% more than Xpert alone.

In a subanalysis, among 97 patients with positive Xpert and/or culture results, 63 (65.0%) were LAM positive.

### Diagnostic yield of algorithms with LAM and without LAM

Table 3 presents the diagnostic yield of algorithms including microscopy or Xpert, with and without LAM. Overall, using LAM increased the diagnostic yield of the algorithm with microscopy by 38.0%, from 60.5% to 98.5%, and the diagnostic yield of the algorithm with Xpert by 34.6%, from 65.4% to 100%. In patients with CD4 below 100 cells/μl, the proportion of patients diagnosed with TB using the 2 algorithms increased by 41.6% and 36.4%, respectively, and in patients with CD4 100–199 cells/μl, by 27.5% and 29.4%, respectively. The yield of each diagnostic tool included in the algorithms is presented in S5 Appendix.

**Table 3:** Diagnostic yield of algorithms including microscopy or Xpert with and without LAM and additional yield of LAM in patients with laboratory-confirmed TB.

Patient group	Algorithm including microscopy*			Algorithm including Xpert**		
	Without LAM	With LAM	Additional yield	Without LAM	With LAM	Additional yield
All patients	60.5 (124/205)	98.5 (202/205)	38.0 (78/205)	65.4 (134/205)	100 (205/205)	34.6 (71/205)
Patients with CD4 < 100 cells/μl	57.1 (88/154)	98.7 (152/154)	41.6 (64/154)	63.6 (98/154)	100 (154/154)	36.4 (56/154)
Patients with CD4 100–199 cells/μl	70.6 (36/51)	98.0 (50/51)	27.5 (14/51)	70.6 (36/51)	100 (51/51)	29.4 (15/51)
Severely ill patients	57.1 (32/56)	98.2 (55/56)	41.1 (23/56)	71.4 (40/56)	100 (56/56)	28.6 (16/56)
Patients with CD4 100–199 cells/μl and not severely ill	69.8 (30/43)	97.7 (42/43)	27.9 (12/43)	67.4 (29/43)	100 (43/43)	32.6 (14/43)

Data given as percent (*n/N*).

*Algorithm including microscopy: clinical signs, microscopy, and chest X-ray, with or without LAM.

**Algorithm including Xpert: clinical signs, Xpert, and chest X-ray, with or without LAM.

LAM, lipoarabinomannan assay; TB, tuberculosis; Xpert, Xpert MTB/RIF assay.

### Risk of mortality in patients solely diagnosed through LAM and not treated

Of the 205 patients with laboratory-confirmed TB, 202 had their vital status ascertained at 6 months and were included in the mortality analysis. Of them, 27 (13.4%) had died. Mortality was 15.6% (26/167) among LAM-positive patients and 2.9% (1/35) among LAM-negative patients (*p =* 0.044). Among the LAM-positive patients, 19 were diagnosed solely through LAM in the early recruitment months in Malawi and were not treated for TB. The mortality rate in this group was 36.8% (7/19), while it was 12.8% (19/148) in LAM-positive patients treated for TB and 2.9% (1/35) in LAM-negative patients treated for TB. In multivariable adjusted analyses (Table 4), LAM-positive patients not diagnosed through other means and not treated for TB had a significantly higher risk of mortality than LAM-positive patients who received anti-TB treatment (adjusted risk ratio 2.57, 95% CI 1.27–5.19, *p =* 0.009). LAM-positive, untreated patients also had a higher risk of mortality than LAM-negative, treated patients (adjusted risk ratio 8.00, 95% CI 1.06–60.51, *p =* 0.044). The results of the multivariable model when missing data were imputed were consistent with the main analysis (S4 Appendix).

**Table 4:** Risk of mortality at 6 months in patients with laboratory-confirmed TB: Univariable and multivariable regression models (*N =* 202).

Variable	*n/N* (%)	RR (95% CI)	*p*-Value	aRR (95% CI)	*p*-Value
**Sex**					
Male	16/119 (13.5)	1			
Female	11/83 (13.3)	0.98 (0.48–2.01)	0.968		
**Age (years)**					
15–29	9/41 (22.0)	1			
30–44	13/122 (10.7)	0.49 (0.22–1.05)	0.067		
≥45	3/37 (8.1)	0.37 (0.11–1.26)	0.112		
**BMI (kg/m**^**2**^**)**					
≥18.5	12/112 (10.7)	1			
17–18.4	9/50 (18.0)	1.68 (0.76–3.73)	0.202		
16–16.9	1/11 (9.1)	0.84 (0.12–5.92)	0.868		
<16	5/27 (18.5)	1.73 (0.67–4.49)	0.261		
**On ART at first consultation**					
No	12/97 (12.4)	1			
Yes	13/101 (12.9)	1.04 (0.50–2.17)	0.916		
**Seriously ill as per clinical exam**					
No	16/146 (11.0)	1			
Yes	11/56 (19.6)	1.79 (0.89–3.62)	0.104		
**CD4 count (cells/μl)**					
≥100	1/50 (2.0)	1			
<100	26/152 (17.1)	8.55 (1.19–61.42)	0.033	7.82 (1.10–55.42)	0.040
**Haemoglobin < 80 g/l**					
No	12/129 (9.3)	1		1	
Yes	11/39 (28.2)	3.03 (1.45–6.32)	0.003	2.12 (1.06–4.23)	0.034
**LAM result and TB treatment**					
Positive and treated	19/148 (12.8)	1		1	
Positive and not treated	7/19 (36.8)	2.87 (1.39–5.91)	0.004	2.57 (1.27–5.19)	0.009
Negative and treated	1/35 (2.9)	0.22 (0.03–1.60)	0.136	0.32 (0.04–2.30)	0.258

aRR, adjusted risk ratio; ART, antiretroviral therapy; BMI, body mass index; LAM, lipoarabinomannan assay; RR, risk ratio; TB, tuberculosis.

## Discussion

We found that under programmatic conditions, including LAM in the diagnostic process for HIV-positive, ambulatory patients with signs and symptoms of TB and CD4 < 200 cells/μl increased considerably the diagnostic yield of algorithms that also included clinical signs, microscopy or Xpert, and chest X-ray. Moreover, in half of the patients with TB, the only positive laboratory test was LAM. These findings were also observed in patients with CD4 100–199 cells/μl. In addition, patients solely diagnosed with LAM who did not initiate TB treatment had an increased risk of mortality compared to those treated for TB.

In our study, LAM was a valuable tool to diagnose TB in HIV-positive patients with CD4 < 200 cells/μl: More than 80% of the patients with laboratory-confirmed TB could have been diagnosed by LAM if the assay had been used diagnostically throughout the study period, and in a high proportion of these cases, other laboratory test results were either negative or not available. A study conducted in various settings in Uganda and South Africa found that LAM could detect more than half of culture-positive TB cases [21]. In Uganda, the sensitivity of Xpert and LAM combined was superior to either test alone and approached the sensitivity of liquid culture testing on sputum [22]. On the other hand, a study conducted in southern Africa found that LAM offered limited value over Xpert or smear microscopy [23]. However, this study only included patients able to produce sputum. In addition, some differences in the diagnostic yield may reflect the pre-test probability, i.e., the prevalence of TB in each study or setting. Although Xpert has a higher sensitivity than LAM, in our study the diagnostic yield of Xpert was lower than that of LAM. This outcome was due to the difficulties in collecting and processing sputum samples: Some patients could not produce sputum or produced only 1 sample, while others did not have an Xpert result due to issues in the transportation of samples to the laboratory or logistical problems in the laboratory. For example, in Malawi, the staff members responsible for collecting sputum samples were also responsible for other community activities and were not always present at the health facility to collect the samples, boxes for the proper transport of samples were often not available at the peripheral centres, and the transport of samples (on motorbike when available or using public vehicles) was not always possible. In addition, the positivity rate of Xpert was low, possibly related to the low quality of some sputum samples (e.g., salivary samples). In the study sites, as is often the case in low-resource settings, sputum induction was not available. In contrast, collecting a urine sample and processing it on LAM on site was an easy procedure and enabled almost all of the patients to have a LAM result. Moreover, the turnaround time was considerably longer for microscopy and Xpert than it was for LAM. Overall, integrating the LAM test into the diagnostic algorithm was easy: It required minimal logistical input, presented little extra workload for the users, and was considered a test that was simple to perform.

Among patients with low CD4 counts, including LAM in the diagnostic algorithm would considerably increase the proportion of patients diagnosed with TB compared to algorithms using either microscopy or Xpert alone. In the context of our study, and in contexts where the delay to receive Xpert results is frequently several days, including the LAM test in the diagnostic algorithm could be useful to start treatment quickly in patients with a positive result while waiting for the Xpert results. In contexts where an Xpert result is available shortly after request, the additional diagnostic value of LAM would be lower. However, performing a LAM test in parallel to Xpert would still offer the advantage of diagnosing patients who cannot produce sputum or who produce a low-quality sputum sample. Nevertheless, a LAM-negative result should not exclude TB, and other examinations should be done. Indeed, in our cohort a proportion of the LAM-negative patients were positive on Xpert or microscopy. Therefore, performing Xpert would allow treating patients not captured by LAM. In addition, performing Xpert in parallel to LAM would be important for identifying patients with drug-resistant TB in contexts with a high prevalence of resistance to rifampicin. LAM can expedite treatment initiation; however, drug susceptibility testing (via at least Xpert) should still be pursued when available.

Current recommendations restrict the use of LAM to ambulatory patients with signs and symptoms of TB and CD4 ≤ 100 cells/μl and are based on studies that have found a lower sensitivity of LAM in patients with CD4 > 100 cells/μl [8,21–24]. However, in our study we found that the LAM test would be valuable in ambulatory patients with signs and symptoms of TB and CD4 100–199 cells/μl. The diagnostic yield of LAM was high in this population. In almost half of the patients with TB, the only positive laboratory test was LAM, and there was a considerable increase in the proportion of patients with TB who were diagnosed when LAM was included in the diagnostic algorithms. In a study in Kenya, LAM alone diagnosed 58% of ambulatory patients with CD4 < 200 cells/μl and microbiologically confirmed pulmonary TB [19]. LAM could therefore be a useful tool to diagnose TB in patients with signs and symptoms of TB and CD4 < 200 cells/μl.

The need for a recent CD4 count to identify patients who would benefit from LAM may be a challenge in some settings, and this would be even more the case if viral load replaces CD4 count for monitoring patients on ART. If a recent or same-day CD4 count is not available and there are no financial or other constraints in using the test, LAM could be performed in all patients with unknown CD4 count, in parallel to requesting sputum-based tests such as Xpert or microscopy. In settings with constraints on performing LAM in all patients with an unknown CD4 count, clinicians and/or programme managers could target severely ill patients and those with possibly advanced disease (e.g., not on ART, virally unsuppressed, or with low BMI).

In our study, as already described by others [13,19,23,25], mortality was higher among LAM-positive patients than among LAM-negative patients. In addition, we observed that LAM-positive patients who were not diagnosed through other tools and who were not treated had an increased risk of mortality compared to treated, LAM-positive patients. In contrast to a study conducted in Myanmar in which the authors concluded that the knowledge of baseline LAM would have been unlikely to avert any of the deaths in the study [26], our findings suggest that in Malawi, knowing the result of LAM during the period when clinicians were blinded to it could have led to a reduction in the mortality in LAM-positive, ambulatory patients with signs and symptoms of TB. This finding is in line with that of the 2 randomised clinical trials that demonstrated that including LAM in the diagnostic algorithm reduces mortality in hospitalised patients with signs and symptoms of TB [15,16].

This study has some limitations. First, some laboratory-confirmed TB diagnoses may have been missed in patients with no Xpert or culture results, which may have led to an overestimation of the proportion of laboratory-confirmed cases diagnosed through LAM. The use of other diagnostic approaches in the diagnostic algorithms, such as Xpert or culture on blood or urine, could have also increased the possibility of detecting TB [6]. Second, the added value of LAM depended on the availability of microscopy or Xpert results, which might vary by setting. Nonetheless, the TB diagnostic conditions in the study sites are similar to those of other resource-limited settings, and we believe that this study reflects real-life practice. Third, previous studies have described that contamination of urine samples and cross-reactivity of the test with non-TB mycobacteria (NTM) may cause false-positive LAM results [20,27]. To reduce the risk of contamination in this study, patients were given careful instructions on how to collect a clean urine sample. A proportion of the LAM-positive patients with sputum culture results had NTM. Although some of them may have had a dual MTB and NTM infection, others might have had disseminated NTM disease [28]; therefore, we cannot exclude the existence of false-positive LAM results.

### Conclusion

LAM has diagnostic value for identifying TB in HIV-positive patients with signs and symptoms of TB and advanced immunodeficiency, including those with a CD4 count of 100–199 cells/μl. In this study, the use of LAM enabled the diagnosis of TB in half of the patients with confirmed TB disease; without LAM these patients would have been missed. The rapid identification and treatment of TB enabled by LAM may decrease overall mortality risk for these patients.

## Supporting information

S1 Appendix STARD checklist. (DOCX)

S2 Appendix Data collection and analysis plan included in the study protocol: Use of LAM test in HIV-infected adults with low CD4 count in programmatic conditions, September 2014. (DOCX)

S3 Appendix Akaike Information Criterion (AIC) and Bayesian information criterion (BIC) for models excluding (complete case analysis) and including the missing values as a separate category. (DOCX)

S4 Appendix Risk of mortality at 6 months in patients with laboratory-confirmed TB: Univariable and multivariable models with imputed missing data. (DOCX)

S5 Appendix Diagnostic yield of the tools composing the algorithms including microscopy or Xpert, with and without LAM, among patients with laboratory-confirmed TB. (DOCX)

## Abbreviations

ART: antiretroviral therapy
BMI: body mass index
LAM: lipoarabinomannan assay
Hb: haemoglobin concentration
MTB: Mycobacterium tuberculosis
NTM: non-tuberculosis mycobacteria
TB: tuberculosis
Xpert: Xpert MTB/RIF assay
