# Supplementary material for: Diagnostic value of the urine lipoarabinomannan assay in HIV-positive, ambulatory patients with CD4 below 200 cells/μl in 2 low-resource settings: A prospective observational study
Source: PLoS Med. 2019 Apr 30;16(4):e1002792. doi: 10.1371/journal.pmed.1002792 (PMC6490904; doi:10.1371/journal.pmed.1002792)
Supplement: S1 Appendix — (DOCX) [file pmed.1002792.s001.docx]

**S1 Appendix: STARD list**

|  | **Section & Topic** | **No** | **Item** | **Reported on page #** |
| --- | --- | --- | --- | --- |
|  |  |  |  |  |
|  | **TITLE OR ABSTRACT** |  |  |  |
|  |  | **1** | Identification as a study of diagnostic accuracy using at least one measure of accuracy  (such as sensitivity, specificity, predictive values, or AUC)  This was not a diagnostic accuracy study. However, we evaluated the diagnostic utility of the LAM test in a real-world setting and in a novel population (patients with a CD4 count below 200), and as such it is stated in the title: “Diagnostic value of the TB-LAM assay in ambulatory HIV-positive patients with CD4 below 200 cells/µL: a prospective observational study.” | Title |
|  | **ABSTRACT** |  |  |  |
|  |  | **2** | Structured summary of study design, methods, results, and conclusions  (for specific guidance, see STARD for Abstracts) | Abstract |
|  | **INTRODUCTION** |  |  |  |
|  |  | **3** | Scientific and clinical background, including the intended use and clinical role of the index test  All these remarks are included in the Background section | Introduction, 1^st^, 2^nd^ and 3^rd^ paragraphs |
|  |  | **4** | Study objectives and hypotheses  The background section finalizes with the study objectives “This study assessed the incremental diagnostic yield of using LAM [...] In addition, we assessed the risk of mortality in patients with a LAM-positive result who were not diagnosed” | Introduction, 4^th^ paragraph |
|  | **METHODS** |  |  |  |
|  | *Study design* | **5** | Whether data collection was planned before the index test and reference standard  were performed (prospective study) or after (retrospective study)  We have stated the prospective study design in the Methods section “We conducted a prospective observational study” | Methods, Section Study design and population, 1^st^ paragraph |
|  | *Participants* | **6** | Eligibility criteria  The inclusion and exclusion criteria are both described in the Methods section “The study population included HIV-positive patients >15 years-old with a CD4 count of less than 200 cell/µl presenting with at least one of the following symptoms of tuberculosis: fever, cough, night sweats, or self-reported weight loss, seeking care at one of our study sites, and who provided written voluntary informed consent. Patients were excluded if they had taken fluoroquinolones or anti-TB drugs in the previous month.” | Methods, Section Study design and population, 1^st^ paragraph |
|  |  | **7** | On what basis potentially eligible participants were identified  (such as symptoms, results from previous tests, inclusion in registry)  This topic is covered in the eligibility criteria section. | Methods, Section Study design and population, 1^st^ paragraph |
|  |  | **8** | Where and when potentially eligible participants were identified (setting, location and dates)  The methods section describes the six facilities included in the study and the study period. | Methods, Section Study design and population, 1^st^ paragraph |
|  |  | **9** | Whether participants formed a consecutive, random or convenience series  This is specified in the Methods section. | Methods, Section Study design and population, 1^st^ paragraph |
|  | *Test methods* | **10a** | Index test, in sufficient detail to allow replication  The main test is detailed in the Study Procedures section “Urine was tested on the Alere Determine TB LAM Ag assay (LAM; Alere, Waltham, MA, USA) on the same day by applying 60μL of fresh urine to the sample pad on the assay strip. Tests were interpreted following the manufacturer’s instructions using the grade 1 (lowest of 4 grades) as the cutoff point for a positive result. ” | Methods, Section Study procedures, 1^st^ paragraph |
|  |  | **10b** | Reference standard, in sufficient detail to allow replication  This point is not applicable to our study as we are not comparing the accuracy of different diagnostic tests. However, all laboratory tests performed are described in the Study procedures section. | Methods, Section Study procedures, 1^st^ paragraph |
|  |  | **11** | Rationale for choosing the reference standard (if alternatives exist)  As in the previous point, this does not apply to our study design. However, the procedures and tests to diagnose TB are described in the Study procedures section. | Methods, Section Study procedures, 1^st^ paragraph |
|  |  | **12a** | Definition of and rationale for test positivity cut-offs or result categories of the index test, distinguishing pre-specified from exploratory  For our study we have not modified the test positivity cut-offs, we have followed the manufacturer’s instructions. | Methods, Section Study procedures, 1^st^ paragraph |
|  |  | **12b** | Definition of and rationale for test positivity cut-offs or result categories of the reference standard, distinguishing pre-specified from exploratory  Not applicable | - |
|  |  | **13a** | Whether clinical information and reference standard results were available to the performers/readers of the index test  The results were available for the study clinician and were indeed used for the patients’ therapeutic management. In Malawi, one of the study sites, LAM test result were was not used for patient management as per the request of the Malawian National TB Program during the first nine months of recruitment, and therefore clinicians were blinded to the test result during this period of time. This information is presented in detailed in the manuscript. | Methods, Section Study procedures, 1^st^ paragraph |
|  |  | **13b** | Whether clinical information and index test results were available to the assessors of the reference standard  This point is not applicable to our study. | - |
|  | *Analysis* | **14** | Methods for estimating or comparing measures of diagnostic accuracy  Our study did not intend to estimate or compare measures of diagnostic accuracy. However, we have specified the methods used to assess the diagnostic value of including the LAM test in the TB diagnosis algorithm in the study population, in the Statistical analyses section. | Methods, Section Statistical analyses, 1^st^ paragraph |
|  |  | **15** | How indeterminate index test or reference standard results were handled  In the study procedures section of our manuscript it is specified how indeterminate results of the TB diagnostic tests were dealt with “Xpert results were reported as positive when MTB was “detected”, negative when MTB was “not detected” and non-available when the test was not performed or the results were reported as “invalid”, “error” or “no result” | Methods, Section Study procedures, 1^st^ paragraph |
|  |  | **16** | How missing data on the index test and reference standard were handled  Please see response to point nb. 15 | Methods, Section Study procedures, 1^st^ paragraph |
|  |  | **17** | Any analyses of variability in diagnostic accuracy, distinguishing pre-specified from exploratory  This type of analyses were not appropriate to our study design. | - |
|  |  | **18** | Intended sample size and how it was determined | - |
|  | **RESULTS** |  |  |  |
|  | *Participants* | **19** | Flow of participants, using a diagram  The flow of participants is presented in Figure 1 of the manuscript. | Figure 1 |
|  |  | **20** | Baseline demographic and clinical characteristics of participants  This information is presented in the main text of the manuscript (beginning of Results section) as well as in Table 1 | Results, Section Patients’ characteristics and Table 1 |
|  |  | **21a** | Distribution of severity of disease in those with the target condition  The severity of disease for the different subpopulations is presented in Table 1. | Table 1 |
|  |  | **21b** | Distribution of alternative diagnoses in those without the target condition  This point is not relevant for the objective of our study since we did not compare the accuracy of different diagnoses assays. | - |
|  |  | **22** | Time interval and any clinical interventions between index test and reference standard  Please see response to point 21b. | - |
|  | *Test results* | **23** | Cross tabulation of the index test results (or their distribution) by the results of the reference standard  Please see response to point 21b. | - |
|  |  | **24** | Estimates of diagnostic accuracy and their precision (such as 95% confidence intervals)  Please see response to point 21b. | - |
|  |  | **25** | Any adverse events from performing the index test or the reference standard  All the tests assessed in these studies are non-invasive, only a urine- and/or sputum-sample were requested, therefore no adverse events were expected or recorded. | - |
|  | **DISCUSSION** |  |  |  |
|  |  | **26** | Study limitations, including sources of potential bias, statistical uncertainty, and generalisability  The limitations of this study are listed at the end of the Discussion section. | Discussion, 7^th^ paragraph |
|  |  | **27** | Implications for practice, including the intended use and clinical role of the index test  These have been included in the Discussion section. | Discussion, 2^nd^ to 6^th^ paragraphs and Conclusions |
|  | **OTHER INFORMATION** |  |  |  |
|  |  | **28** | Registration number and name of registry  This study has not been registered as it is not a diagnostic accuracy study. | - |
|  |  | **29** | Where the full study protocol can be accessed  The data collection and analysis plan of the study protocol has been included as a Supporting Information file and has been cited in the Methods section. | Supporting information |
|  |  | **30** | Sources of funding and other support; role of funders  This study has been funded by Médecins Sans Frontières. The information regarding its role has been stated in the Financial Disclosure Statement | Financial Disclosure Statement |
|  |  |  |  |  |
