# Supplementary material for: Diagnostic value of the urine lipoarabinomannan assay in HIV-positive, ambulatory patients with CD4 below 200 cells/μl in 2 low-resource settings: A prospective observational study
Source: PLoS Med. 2019 Apr 30;16(4):e1002792. doi: 10.1371/journal.pmed.1002792 (PMC6490904; doi:10.1371/journal.pmed.1002792)
Supplement: S2 Appendix — (DOCX) [file pmed.1002792.s002.docx]

**S2 Appendix: Data collection and analysis plan included in the study protocol: Use of LAM test in HIV infected adults with low CD4 count in programmatic conditions****, September 2014.**

Data collection:

The following information concerning the patients will be collected: demographic information, CD4 count, ART start and date, ART regimen, clinical data at baseline, LAM test results, microscopy results, Xpert MTB/RIF results, radiological findings, culture results (if applicable) clinical condition, suspicion of TB, type of antibiotic treatment (name, duration, dose), decision to initiate TB treatment (date), type of TB (pulmonary or extrapulmonary), TB regimen, clinical situation and outcomes at 2 weeks, 2 months and 6 months, date of death if deceased, results of tracing (if applicable).

The following information concerning the clinic will be collected: number of clinicians performing LAM and number of tests performed per day; time per day and per person to perform LAM; space and physical organisation of the clinic to accommodate LAM test; equipment and material needed to perform LAM; cost of the activity (training, human resources, material, equipment); challenges faced during implementation, LAM test double reading result.

Analysis plan – end points:

- Proportion of confirmed TB patients started on TB treatment due to a LAM positive result among TB suspects HIV infected
- Proportion of suspected TB patients with LAM negative results but confirmed TB diagnosis started on TB treatment.
- Proportion of suspected TB patients with LAM negative results and not confirmed TB diagnosis started on TB treatment.
- Reasons to start TB treatment in TB suspects HIV infected
- LAM test results by immunological status and clinical condition
- Outcomes at 2 weeks, 2 months and 6 months of patients according to the LAM test result
- LAM test reading agreement
- Time from beginning of consultation to management decision
- Time to perform LAM test
- Number of clinicians/nurses/lab technicians performing LAM test
- Number of LAM tests performed per day
- Space and physical organisation of the clinic to accommodate LAM test
- Equipment and material used to perform LAM
- Health service direct cost: human resources, material, equipment, training
- Challenges faced during implementation.
