# Supplementary material for: Diagnostic value of the urine lipoarabinomannan assay in HIV-positive, ambulatory patients with CD4 below 200 cells/μl in 2 low-resource settings: A prospective observational study
Source: PLoS Med. 2019 Apr 30;16(4):e1002792. doi: 10.1371/journal.pmed.1002792 (PMC6490904; doi:10.1371/journal.pmed.1002792)
Supplement: S3 Appendix — (DOCX) [file pmed.1002792.s003.docx]

**S3 Appendix: Akaike Information Criterion (AIC) and Bayesian information criterion (BIC) for models excluding (complete-case analysis) and including the missing values as a separate category**

**Table S3-A: Akaike Information Criterion (AIC) and Bayesian information criterion (BIC) for restricted models excluding the missing values (complete-case analysis).**

|  | **Age** | **Seriously ill** | **Haemoglobin** | **CD4 count** | **LAM result & TB treatment** | **AIC** | **BIC** |
| --- | --- | --- | --- | --- | --- | --- | --- |
| **Model 0** | 1 | 1 | 1 | 1 | 1 | 114.3231 | 139.219 |
| **Model 1** | 1 | 1 | 1 | 1 | 0 | 119.1353 | 137.8073 |
| **Model 2** | 1 | 1 | 1 | 0 | 1 | 118.1037 | 139.8876 |
| **Model 3** | 1 | 1 | 0 | 1 | 1 | 115.677 | 137.4609 |
| **Model 4** | 1 | 0 | 1 | 1 | 1 | 113.9203 | 135.7043 |
| **Model 5** | 0 | 1 | 1 | 1 | 1 | 112.3881 | 131.06 |
| **Model 6** | 1 | 1 | 1 | 0 | 0 | 123.4299 | 138.9898 |
| **Model 7** | 1 | 1 | 0 | 1 | 0 | 120.8937 | 136.4537 |
| **Model 8** | 1 | 0 | 1 | 1 | 0 | 118.4233 | 133.9832 |
| **Model 9** | 0 | 1 | 1 | 1 | 0 | 117.8196 | 130.2676 |
| **Model 10** | 1 | 1 | 0 | 0 | 1 | 118.5128 | 137.1847 |
| **Model 11** | 1 | 0 | 1 | 0 | 1 | 117.9686 | 136.6406 |
| **Model 12** | 0 | 1 | 1 | 0 | 1 | 118.2512 | 133.8112 |
| **Model 13** | 1 | 0 | 0 | 1 | 1 | 115.7002 | 134.3721 |
| **Model 14** | 0 | 1 | 0 | 1 | 1 | 115.3467 | 130.9067 |
| **Model 15** | 0 | 0 | 1 | 1 | 1 | 112.0093 | 127.5693 |
| **Model 16** | 1 | 1 | 0 | 0 | 0 | 125.3453 | 137.7933 |
| **Model 17** | 1 | 0 | 1 | 0 | 0 | 122.8454 | 135.2933 |
| **Model 18** | 0 | 1 | 1 | 0 | 0 | 123.2471 | 132.583 |
| **Model 19** | 1 | 0 | 0 | 1 | 0 | 120.6832 | 133.1311 |
| **Model 20** | 0 | 1 | 0 | 1 | 0 | 121.4869 | 130.8228 |
| **Model 21** | 0 | 0 | 1 | 1 | 0 | 116.9575 | 126.2935 |
| **Model 22** | 1 | 0 | 0 | 0 | 1 | 118.8685 | 134.4285 |
| **Model 23** | 0 | 1 | 0 | 0 | 1 | 120.2497 | 132.6976 |
| **Model 24** | 0 | 0 | 0 | 1 | 1 | 115.4089 | 127.8569 |

Note: Numbers “1” indicate those variables that were included in the model. Numbers “0” indicate variables that were not included in the models.

**Table S3-B: Akaike Information Criterion (AIC) and Bayesian information criterion (BIC) for models that included missing values in Haemoglobin count as a separate category.**

|  | **Age** | **Seriously ill** | **Haemoglobin** | **CD4 count** | **LAM result & TB treatment** | **AIC** | **BIC** |
| --- | --- | --- | --- | --- | --- | --- | --- |
| **Model 0** | 1 | 1 | 1 | 1 | 1 | 140.8688 | 170.5536 |
| **Model 1** | 1 | 1 | 1 | 1 | 0 | 146.646 | 169.7342 |
| **Model 2** | 1 | 1 | 1 | 0 | 1 | 146.5496 | 172.9361 |
| **Model 3** | 1 | 1 | 0 | 1 | 1 | 141.2069 | 164.2952 |
| **Model 4** | 1 | 0 | 1 | 1 | 1 | 139.9377 | 166.3243 |
| **Model 5** | 0 | 1 | 1 | 1 | 1 | 138.0508 | 161.139 |
| **Model 6** | 1 | 1 | 1 | 0 | 0 | 152.1292 | 171.9191 |
| **Model 7** | 1 | 1 | 0 | 1 | 0 | 148.2075 | 164.6991 |
| **Model 8** | 1 | 0 | 1 | 1 | 0 | 145.5281 | 165.318 |
| **Model 9** | 0 | 1 | 1 | 1 | 0 | 144.1043 | 160.5959 |
| **Model 10** | 1 | 1 | 0 | 0 | 1 | 145.6468 | 165.4367 |
| **Model 11** | 1 | 0 | 1 | 0 | 1 | 145.9785 | 169.0667 |
| **Model 12** | 0 | 1 | 1 | 0 | 1 | 144.4107 | 164.2006 |
| **Model 13** | 1 | 0 | 0 | 1 | 1 | 140.401 | 160.1909 |
| **Model 14** | 0 | 1 | 0 | 1 | 1 | 139.0456 | 155.5372 |
| **Model 15** | 0 | 0 | 1 | 1 | 1 | 137.3661 | 157.156 |
| **Model 16** | 1 | 1 | 0 | 0 | 0 | 153.3455 | 166.5388 |
| **Model 17** | 1 | 0 | 1 | 0 | 0 | 151.2463 | 167.7379 |
| **Model 18** | 0 | 1 | 1 | 0 | 0 | 150.0545 | 163.2477 |
| **Model 19** | 1 | 0 | 0 | 1 | 0 | 147.0411 | 160.2343 |
| **Model 20** | 0 | 1 | 0 | 1 | 0 | 146.5458 | 156.4408 |
| **Model 21** | 0 | 0 | 1 | 1 | 0 | 143.1335 | 156.3267 |
| **Model 22** | 1 | 0 | 0 | 0 | 1 | 145.4773 | 161.9689 |
| **Model 23** | 0 | 1 | 0 | 0 | 1 | 144.4455 | 157.6388 |
| **Model 24** | 0 | 0 | 0 | 1 | 1 | 138.6786 | 151.8719 |

Note: Numbers “1” indicate those variables that were included in the model. Numbers “0” indicate variables that were not included in the models.
