# Supplementary material for: Diagnostic value of the urine lipoarabinomannan assay in HIV-positive, ambulatory patients with CD4 below 200 cells/μl in 2 low-resource settings: A prospective observational study
Source: PLoS Med. 2019 Apr 30;16(4):e1002792. doi: 10.1371/journal.pmed.1002792 (PMC6490904; doi:10.1371/journal.pmed.1002792)
Supplement: S4 Appendix — (DOCX) [file pmed.1002792.s004.docx]

**S4 Appendix: Risk of mortality at 6 months in patients with laboratory-confirmed TB: univariable and multivariable models** **with imputed missing data**

**Table S4: Risk of mortality at 6 months in patients with laboratory-confirmed TB: univariable and multivariable models** **with imputed missing data**

|  | **RR** | **(95% CI)** | | | **p-value** | **aRR** | **(95% CI)** | | | **p-value** |
| --- | --- | --- | --- | --- | --- | --- | --- | --- | --- | --- |
| **Sex** |  |  |  |  |  |  |  |  |  |  |
| Male | 1 |  |  |  |  | - |  |  |  |  |
| Female | 0.98 | (0.43 | - | 2.24) | 0.968 |  |  |  |  |  |
| **Age (in years)** |  |  |  |  |  |  |  |  |  |  |
| 15-29 | 1 |  |  |  |  | - |  |  |  |  |
| 30-44 | 0.42 | (0.17 | - | 1.08) | 0.073 |  |  |  |  |  |
| >=45 | 0.31 | (0.08 | - | 1.26) | 0.103 |  |  |  |  |  |
| **BMI (kg/m2)** |  |  |  |  |  |  |  |  |  |  |
| >=18.5 | 1 |  |  |  |  | - |  |  |  |  |
| 17-18.4 | 1.83 | (0.72 | - | 4.67) | 0.207 |  |  |  |  |  |
| 16-16.9 | 0.83 | (0.10 | - | 7.09) | 0.867 |  |  |  |  |  |
| <16 | 1.89 | (0.61 | - | 5.93) | 0.273 |  |  |  |  |  |
| **On-ART at first consultation** |  |  |  |  |  |  |  |  |  |  |
| No | 1 |  |  |  |  | - |  |  |  |  |
| Yes | 1.05 | (0.45 | - | 2.42) | 0.916 |  |  |  |  |  |
| **Seriously ill** |  |  |  |  |  |  |  |  |  |  |
| No | 1 |  |  |  |  | - |  |  |  |  |
| Yes | 1.99 | (0.86 | - | 4.60) | 0.109 |  |  |  |  |  |
| **CD4 count (cells/µL)** |  |  |  |  |  |  |  |  |  |  |
| >=100 | 1 |  |  |  |  | 1 |  |  |  |  |
| <100 | 10.11 | (1.34 | - | 76.55) | 0.025 | 11.52 | (1.39 | - | 95.65) | 0.024 |
| **Haemoglobin <8 gr/dl** |  |  |  |  |  |  |  |  |  |  |
| No | 1 |  |  |  |  | 1 |  |  |  |  |
| Yes | 3.12 | (1.28 | - | 7.64) | 0.013 | 2.74 | (1.05 | - | 7.12) | 0.039 |
| **LAM result & TB treatment** |  |  |  |  |  |  |  |  |  |  |
| Positive & Treated | 1 |  |  |  |  | 1 |  |  |  |  |
| Positive & Not treated | 3.96 | (1.39 | - | 11.31) | 0.010 | 4.91 | (1.56 | - | 15.47) | 0.007 |
| Negative & Treated | 0.20 | (0.03 | - | 1.55) | 0.123 | 0.30 | (0.04 | - | 2.41) | 0.258 |
