# Supplementary material for: Diagnostic value of the urine lipoarabinomannan assay in HIV-positive, ambulatory patients with CD4 below 200 cells/μl in 2 low-resource settings: A prospective observational study
Source: PLoS Med. 2019 Apr 30;16(4):e1002792. doi: 10.1371/journal.pmed.1002792 (PMC6490904; doi:10.1371/journal.pmed.1002792)
Supplement: S5 Appendix — (DOCX) [file pmed.1002792.s005.docx]

**S5 Appendix: Diagnostic yield of the tools comprised in the algorithms including microscopy or Xpert, with and without LAM, among patients with laboratory-confirmed TB**

We evaluated two algorithms: the first one included clinical exam, smear microscopy and chest X-ray, the second one included clinical exam, Xpert MTB/RIF in sputum and chest X-ray. For each algorithm, we assessed the diagnostic yield of each tool in algorithms that comprised or not the LAM, among patients with a laboratory-confirmed TB. The yield of each diagnostic tool was calculated sequentially using the order in which the results were available to the clinicians. The diagnostic yield was assessed from the perspective of the patient’s management with the objective of replicating real life decision making. A patient was considered as diagnosed through the clinical exam if the clinician would have empirically treated the patient based on the clinical exam findings alone and prior to receiving any other diagnostic tool result. To assess this, clinicians were asked in real time, immediately after performing the clinical exam, what their diagnosis and treatment decision would be. Similarly, a patient was considered as diagnosed through the chest X-ray if the clinician interpreted the X-ray as very suggestive of TB and the patient was not diagnosed through any other tool included in the algorithm. A patient was considered as diagnosed through LAM, microscopy or Xpert when the results of the respective tests were positive in the sequence used in the algorithm. As a result, the yield of each of the tools assessed decreased significantly when LAM was included in the algorithm due to the high proportion of patients diagnosed through this test.

**Table S5: Diagnostic yield of each of the tools comprised in different algorithms including microscopy or Xpert, with and without LAM, in patients with laboratory-confirmed TB**

|  | **Algorithms including microscopy** | | | | **Algorithms including Xpert** | | |
| --- | --- | --- | --- | --- | --- | --- | --- |
|  | **Without LAM** | | **With LAM** | | **Without LAM** | | **With LAM** |
|  | Clinic > Microscopy > X-ray | | LAM > Clinic > Microscopy > X-ray | | Clinic > Xpert > X-ray | | LAM > Clinic > Xpert > X-ray |
|  | % (n/N) | | % (n/N) | | % (n/N) | | % (n/N) |
| **All patients** | |  | | | |  | |
| - LAM | - | | 82.4 (169/205) | | - | | 82.4 (169/205) |
| - Clinical signs | 31.7 (65/205) | | 7.3 (15/205) | | 31.7 (65/205) | | 7.3 (15/205) |
| - Microscopy | 24.4 (50/205) | | 7.3 (15/205) | | - | | - |
| - Xpert on sputum | - | | - | | 29.3 (60/205) | | 8.8 (18/205) |
| - X-ray | 4.4 (9/205) | | 1.5 (3/205) | | 4.4 (9/205) | | 1.5 (3/205) |
| **Patients with CD4<100** | |  | | | |  | |
| - LAM | - | | 86.4 (133/154) | | - | | 86.4 (133/154) |
| - Clinical signs | 31.8 (49/154) | | 7.8 (12/154) | | 31.8 (49/154) | | 7.8 (12/154) |
| - Microscopy | 21.4 (33/154) | | 3.3 (5/154) | | - | | - |
| - Xpert on sputum | - | | - | | 31.8 (42/154) | | 3.9 (6/154) |
| - X-ray | 3.9 (6/154) | | 1.3 (2/154) | | 4.6 (7/154) | | 2.0 (3/154) |
| **Patients with CD4 100-199** | |  | | | |  | |
| - LAM | - | | 70.6 (36/51) | | - | | 70.6 (36/51) |
| - Clinical signs | 31.4 (16/51) | | 5.9 (3/51) | | 31.4 (16/51) | | 5.9 (3/51) |
| - Microscopy | 33.3 (17/51) | | 19.6 (10/51) | | - | | - |
| - Xpert on sputum | - | | - | | 35.3 (18/51) | | 23.5 (12/51) |
| - X-ray | 5.9 (3/51) | | 2.0 (1/51) | | 3.9 (2/51) | | 0.0 (0/51) |
| **Patients severely ill** | |  | | | |  | |
| - LAM | - | | 89.3 (50/56) | | - | | 89.3 (50/56) |
| - Clinical signs | 41.1 (23/56) | | 7.1 (4/56) | | 41.1 (23/56) | | 7.1 (4/56) |
| - Microscopy | 16.1 (9/56) | | 1.8 (1/56) | | - | | - |
| - Xpert on sputum | - | | - | | 30.4 (17/56) | | 3.6 (2/56) |
| - X-ray | 0.0 (0/56) | | 0.0 (0/56) | | 0.0 (0/56) | | 0.0 (0/56) |
| **Patients with CD4 100-199 not severely ill** | | | |  | | | |
| - LAM | - | | 69.8 (30/43) | | - | | 69.8 (30/43) |
| - Clinical signs | 25.6 (11/43) | | 4.7 (2/43) | | 25.6 (11/43) | | 4.7 (2/43) |
| - Microscopy | 37.2 (16/43) | | 20.9 (9/43) | | - | | - |
| - Xpert on sputum | - | | - | | 37.2 (16/43) | | 25.6 (11/43) |
| - X-ray | 7.0 (3/43) | | 2.3 (1/43) | | 4.7 (2/43) | | 0.0 (0/43) |
